# Olive Oil Produced from Olives Stored under CO_2_ Atmosphere: Volatile and Physicochemical Characterization

**DOI:** 10.3390/antiox12010030

**Published:** 2022-12-24

**Authors:** Vassilis Athanasiadis, Theodoros Chatzimitakos, Eleni Bozinou, Dimitris P. Makris, Vassilis G. Dourtoglou, Stavros I. Lalas

**Affiliations:** 1 Department of Food Science and Nutrition, University of Thessaly, 43100 Karditsa, Greece; 2 Department of Wine, Vine, and Beverage Sciences, School of Food Science, University of West Attica, 12243 Athens, Greece

**Keywords:** antioxidants, carbon dioxide, cold pressed, extra virgin olive oil, fatty acids, HS-SPME/GC-MS, modified atmospheres, olives, sensory evaluation, volatile and bioactive compounds

## Abstract

In this study, an alternative debittering technique for olives, invented and patented by Prof. Vassilis Dourtoglou, was employed. Olive fruits (*Olea europaea* cv. Megaritiki) were stored under CO_2_ atmosphere immediately after harvest for a period of 15 days. After the treatment, a sensory evaluation between the olives stored under CO_2_ and those stored under regular atmospheric conditions (control) was performed. Additionally, the CO_2_-treated olives were used for the cold press of olive oil production. The volatile profile of the olive oil produced was analyzed using headspace solid-phase microextraction (HS-SPME) and gas chromatography coupled to mass spectrometry (GC-MS). A total of thirty different volatile compounds were detected. The volatile characteristics of olive oil are attributed, among others, to aldehydes, alcohols, esters, hydrocarbons, alkanes, and terpenes. The volatile compounds’ analysis showed many differences between the two treatments. In order to compare the volatile profile, commercial olive oil was also used (produced from olives from the same olive grove with a conventional process in an industrial olive mill). The antioxidant activity, the content of bioactive compounds (polyphenols, α-tocopherol, carotenoids, and chlorophylls), and the fatty acids’ profile were also determined. The results showed that the oil produced from CO_2_-treated olives contains different volatile components, which bestow a unique flavor and aroma to the oil. Moreover, this oil was found comparable to extra virgin olive oil, according to its physicochemical characteristics. Finally, the enhanced content in antioxidant compounds (i.e., polyphenols) not only rendered the oil more stable against oxidation but also better for human health. The overall quality of the olive oil was enhanced and, as such, this procedure holds great promise for future developments.

## 1. Introduction

Table olives are an integral part of the Mediterranean diet. Their consumption is considered beneficial, since it includes a well-balanced amount of fats, the majority of which is the monounsaturated oleic acid [1]. Olive fruit’s flesh has high nutritional and biological value because of its content in essential amino and fatty acids, vitamins (especially α-tocopherol), and many minerals. Triacylglycerols make up most of olive oil (97–98%), followed by free fatty acids (oleic, linoleic, palmitic, and others), mono- and diacylglycerols, and a variety of lipids including hydrocarbons (squalene), sterols, aliphatic alcohols, tocopherols (primarily α-tocopherol), and pigments (chlorophylls and carotenoids). Some of these substances contribute to the oil’s aroma [2,3,4,5]. Aroma is an important parameter that shapes the consumers’ preferences. Therefore, much emphasis is being placed on the aroma of olive oil, which depends on factors, such as ripening, variety, climate, and processing conditions [6]. During the processing of olives for olive oil production, many changes in its composition take place. One important change is that part of oleuropein (the main polyphenol of olives) is partly hydrolyzed, producing other substances that participate in the characteristic taste and aroma of the oil [7]. 

Another process that takes place during the processing of olives to produce oil is the oxidation of phenolic compounds [8,9], which results in the creation of substances that bestow olive oil’s unique aroma and flavor [10]. Different volatile compounds (VCs) such as aldehydes, alcohols, esters, hydrocarbons, ketones, furans, and other unidentified VCs are responsible for the development of its unique flavor [11]. Such compounds can also be formed in the olive fruit by enzymatic activity [12]. The biogenic mechanisms of olive fruit, particularly the lipoxygenase (LOX) pathways and the metabolism of fatty acids or amino acids are responsible for the production of the VCs found in high-quality virgin olive oil [13,14]. In addition, the LOX pathway is the main mechanism via which the most significant volatile components of olive oil are produced [15]. Products from the LOX pathway, such as aldehydes, alcohols, and their esters, make up the majority (~80%) of the VCs in high-quality virgin olive oils. These compounds have six straight-chain carbons (C_6_) and five straight-chain carbons (C_5_) [16,17,18,19]. As a result, the relative activity of the enzymes in the LOX pathway, which is affected by different factors such as the type of olives and their maturation level or the conditions under which the oil is extracted, determines the aroma of olive oil [18,19]. As stated above, VCs are crucial to the quality of olive oil, whether they are present in high or low concentrations. In order to understand the formation and degradation of the volatile compounds that significantly contribute to the aroma of olive oil, VCs that exist in olive oil below their sensory threshold may be important quality indicators [20]. To determine their relative contribution to the production of the olive oil aroma, it is necessary to characterize each volatile class in its components [21].

In 2005, Dourtoglou V. patented for the first time a debittering process of olives using a CO_2_ atmosphere or CO_2_/N_2_/O_2_ atmosphere [22]. Despite the fact that CO_2_ atmosphere had been previously used for storage, it was found that this procedure holds great promise for the debittering and, thus, for the processing of table olives [23]. It was found that the phenolic composition of freshly harvested olives can change significantly under a CO_2_ atmosphere, along with their sensory attribute. Therefore, if put to good use, this procedure can pave the way for controlling the content of bioactive compounds in olives [24]. To the best of our knowledge, there are no reports regarding the flavor and physicochemical properties of olive oil produced from olives pre-processed in a CO_2_ atmosphere. Therefore, the objective of the current study was to identify the quality (sensory) characteristics, the VCs, and some physicochemical parameters of cold-pressed oil. To this end, olives of the Megaritiki variety were used, after post-harvest storage under CO_2_ atmosphere, without the use of chemicals (e.g., NaOH and NaCl).

## 2. Materials and Methods

### 2.1. Plant Material

Green olives (*Olea europaea*) of the Megaritiki variety were harvested from olive groves in the area of Stylida (Phthiotis prefecture, Greece). The olives were randomly picked from the trees at the industrial optimal ripening stage (in mid-October), according to their skin color (green).

### 2.2. Post-Harvest Treatment

**Figure 1:**
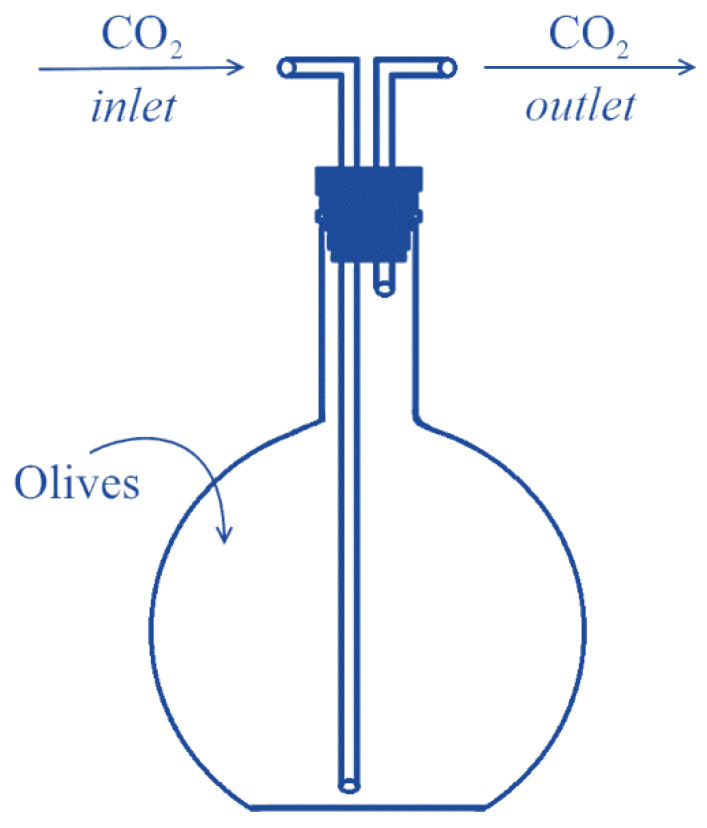
Schematic representation of the glass jar used for the post-harvest treatment of olives. The CO_2_ gas was added to the bottom of the glass jar, via a glass tube, to ascertain saturation of the inner of the jar with CO_2_.

The process was based on a previously reported patent (patent number: GR20050100482A) by Dourtoglou [22]. Fruits were cleaned and their leaves were removed before being separated into two batches of 1 Kg each. The first batch was placed in a glass jar, under a CO_2_ atmosphere, as shown in Figure 1. The gas flow (1 L/min) was continuous for 15 days. At the CO_2_ outlet, the gas was led into a potassium hydroxide solution, so that CO_2_ emissions could be minimized. The other batch, which served as control, was left in the air, in a similar glass jar. In both cases, the treatment was carried out at 23 ± 2 °C and 90–95% relative humidity. The olives were weighed before and after the procedure.

### 2.3. Sensory Evaluation

In order to evaluate the sensory characteristics of the olives, they were placed in plastic dishes [7]. Sensory evaluation was conducted at the Sensory Evaluation Laboratory of the Department of Food Science and Nutrition (University of Thessaly) by twelve panelists. A three-digit code was used to encode every sample. Using a numerical range of 1–9 (1 = not acceptable, 9 = highly acceptable), the panelists assessed the overall acceptability of each sample (considering any off-flavor and undesirable taste), as well as its bitterness (1 = no bitterness, 9 = extremely bitter). Additionally, panelists were asked to state their overall preference.

### 2.4. Olive Oil Production

The production of olive oil was carried out by a cold mechanical press. Initially, the stone from debittered olives was removed manually. Then, the fruits were crushed into pulp using a ball mill (Planetary Mono Mill Pulverisette 6 classic line, Fritsch GmbH, Idar-Oberstein, Germany). The fruit pulp was placed in a cotton fabric bag. The bag was immediately pressed with a hydraulic press (Atlas Manual Hydraulic Press, Specac Ltd, Kent, UK) in order to extract the oil. The extracted liquid was centrifuged using a Digicen 20-R (Orto Alresa, Madrid, Spain) for 10 min at 4500 rpm in order to separate the oil. A commercially available olive oil, produced from olives from the same olive grove using a conventional extraction procedure in an industrial olive mill, was also examined for comparison purposes.

### 2.5. Volatile Compounds (VCs) Analysis

The VCs of the samples were extracted under the principle of headspace solid-phase microextraction, according to a previous study [13]. A solid phase microextraction fiber (Supelco, Bellefonte, PA, USA) coated with a layer of divinylbenzene/carboxen/polydimethylsiloxane (DVB/CAR/PDMS) was used. The fiber was preconditioned (1 h at 260 °C) according to the manufacturer’s recommendations before usage. For the extraction of the VCs, 3 g of olive oil was put in a 5 mL glass vial and sealed with an appropriate PTFE/silicone septum. The vial was placed in a water bath at 40 °C for 10 min for equilibration. The fiber was inserted in the headspace of the vial and extraction was carried out for 1 h, under constant stirring of the sample at 200 rpm. After the extraction was completed, the fiber was removed and placed in the injector of the Gas Chromatography coupled with Mass Spectrometry (GC-MS) system. The instrument was an Agilent Technologies (Santa Clara, CA, USA) Gas Chromatograph model 7890A coupled to a mass detector (model 5975C, Agilent Technologies, Santa Clara, CA, USA) and a capillary column Omegawax (30 m × 320 μm × 0.25 μm) (Supelco, Bellefonte, PA, USA). Helium was used as a carrier gas and the flow rate was set at 1 mL/min. Injector temperature was set at 230 °C and injections were made in splitless mode. The column was maintained at 30 °C for 30 min and heated to 220 °C at a rate of 2 °C/min for 10 min. The conditions of the detector were as follows: source temperature 230 °C; quadrupole temperature 150 °C; acquisition mode electron impact (EI 69.9 eV) and mass range *m/z* 29–350. All analyses were carried out in triplicate. The spectra were evaluated using Agilent Chemstation (version B.03.02). Identification of the compounds was based on comparing the individual mass spectra to those found in the Wiley W8N08 database (Wiley, New York, USA). Using a normalization procedure, the percentage composition of the samples was calculated from the GC peak areas (without correction factors). Results were expressed as mean values of the three replicate analyses.

### 2.6. Physicochemical Characteristics Analysis

#### 2.6.1. Acidity Value

Determination of free fatty acids (FFAs) was carried out according to Commission Regulation (EEC) No 2568/91 (Annex II) [25].

#### 2.6.2. Ultraviolet Spectrophotometric Examination (K_232_, K_270_, ΔK)

Oil samples were examined spectrophotometrically in the ultraviolet spectrum, according to Commission Implementing Regulation No 299/2013 (Annex Ι) [26] using a Shimadzu UV-1700 UV/Vis spectrophotometer (Kyoto, Japan). Specifically, 0.25 g of oil was mixed with 25 mL cyclohexane in a tube and after thorough mixing, the absorbance was recorded at 232 nm and 270 nm. 

#### 2.6.3. Colorimetry

The color of the oil samples was determined using a colorimeter (Lovibond CAM-System 500, The Tintometer Ltd, Amesbury, UK). A total of 25 mL of oil was added in a 50 mL glass beaker and the beaker was transferred in the colorimeter for CIELAB color determination. Two color coordinates, a* and b*, and the psychometric index of lightness, L*, were defined. The colorimetric parameters Chroma $\left(C_{ab}^{*}\right)$ and hue angle ($h_{ab}^{o}$) were also determined as follows:

(1) $$C_{ab}^{*}=\sqrt{{\left(a^{*}\right)}^{2}+{\left(b^{*}\right)}^{2}}$$

(2) $$h_{ab}^{o}=\arctan\left(\frac{b^{*}}{a^{*}}\right)$$

#### 2.6.4. Pigments

The pigment content of the samples (carotenoids and chlorophylls) were measured according to a previous protocol [27]. A total of 5 mL of cyclohexane was mixed with 1.5 g of olive oil in a tube. The absorbance of the sample was measured at 470 nm (*A*_470_) and 670 nm (*A*_670_). The extinction coefficients used had the values of *E_o_* = 2,000 for lutein and *E_o_* = 613 for pheophytin α. Pigment content was calculated as follows:

(3) $$C_{\mathrm{carotenoids}\;}(\mathrm{mg}/\mathrm{Kg}\;\mathrm{of}\;\mathrm{oil})=\frac{A_{470}{\;\times \;10}^{6}}{2000\;\times \;100\;\times \;s}$$

(4) $$C_{\mathrm{chlorophylls}\;}(\mathrm{mg}/\mathrm{Kg}\;\mathrm{of}\;\mathrm{oil})=\frac{A_{670}{\;\times \;10}^{6}}{613\;\times \;100\;\times \;s}$$

where *s*: the thickness of the quartz cells (in cm). 

The amount of lutein and pheophytin α per Kg of oil, respectively, was used to represent the carotenoid and chlorophyll concentrations. Three replicates were carried out for all the samples. Total pigments (*C*_carotenoids_ + *C*_chlorophylls_) and the ratio chlorophylls/carotenoids (*R*_chl/car_) were also determined.

#### 2.6.5. Total Polyphenol Content

The extraction of total polyphenols from olive oil was carried out according to a previous report [28]. Olive oil samples (1 g) were dissolved in 2 mL of *n*-hexane before being extracted with 2 mL of a 60:40 (*v/v*) methanol/water solution. The mixture was vigorously stirred using vortex, and then the sample was centrifuged at 4500 rpm for 5 min. The soluble (polar) fraction of olive oil was obtained and used for the determination of total polyphenols, according to a previous report [29].

#### 2.6.6. A-Tocopherol Content

The determination of the α-tocopherol content was based on a previous study [7]. The analysis was done using a Shimadzu CBM-20A (Shimadzu Europa GmbH, Duisburg, Germany) high-performance liquid chromatograph (HPLC), equipped with a SIL-20AC autosampler and a CTO-20AC column oven. Shimadzu’s RF-10AXL fluorescence detector was used for detection, with the excitation and emission wavelengths set to 294 and 329 nm, respectively. A Waters μ-Porasil column (125 Å, 10 μm, 3.9 mm × 300 mm; Waters Corp., Milford, MA, USA) was used as a stationary phase. The sample was prepared as follows: 0.25 g of oil sample were placed in a 5 mL volumetric flask and *n*-hexane was added. After vigorous stirring, a 20 μL sample was injected into the HPLC system. The mobile phase consisted of *n*-hexane/2-propanol/absolute ethanol (97.5:2:0.5, *v/v/v*) and the flow rate was set at 1 mL/min. The amount of α-tocopherol in olive oil (α-TC) was calculated as mg of α-tocopherol per Κg of oil.

#### 2.6.7. Rancimat Method

The oxidation stability of the olive oils was carried out by the Rancimat method as previously reported [7]. The reaction vessels of the Rancimat 743 (Metrhom LTD, Herisau, Switzerland) were filled with 3 g of each sample. Samples were heated at 90 °C and the airflow was set at 15 L/h. The induction period (in hours) was calculated automatically.

#### 2.6.8. Fatty Acid Composition

The fatty acid composition of the samples was carried out by preparing the respective fatty acid methyl esters (FAMEs), according to the Commission Regulation (EC) No 796/2002 (Annex XB) [30]. Analysis of FAMEs was carried out, as described previously [31]. An Agilent Technologies Gas Chromatograph model 7890A (Santa Clara, CA, USA), equipped with a capillary column Omegawax (30 m × 320 μm × 0.25 μm) (Supelco). Helium was the carrier gas at a flow rate of 1.4 mL/min. The column temperature program was initially kept constant for 5 min at 70 °C, then increased with a rate of 20 °C/min up to 160 °C, then with a rate of 4 °C/min up to 200 °C and with a rate of 5 °C/min up to 240 °C. Temperatures for the injector and flame ionization detector (FID) were kept at 240 and 250 °C, respectively. Air flow was set at 450 mL/min, hydrogen flow at 50 mL/min, and helium flow at 50 mL/min for makeup. Samples of 1.0 μL were injected into splitless mode. Identification of the compounds was carried out by comparing the retention times with that of standard compounds [Supelco 37-Component FAME Mix reference standard (Supelco)]. Using the normalization procedure, the percentage composition of the samples was calculated from the GC peak areas (without correction factors). Results were expressed as mean values of three replicate analyses.

### 2.7. Statistical Analysis

The average and standard deviation (SD) (in parenthesis) of three replicate analyses were used to express the results. The normality of the distribution of the results was examined with the Shapiro–Wilk test. Analysis of variance (ANOVA) was used to determine whether the differences between the mean values were statistically significant; a significance level of *p* < 0.05 was used. All statistical analyses were carried out using SPSS (version 26) (SPSS Inc., Chicago, IL, USA) software.

## 3. Results and Discussion

### 3.1. Color Change

**Figure 2:**
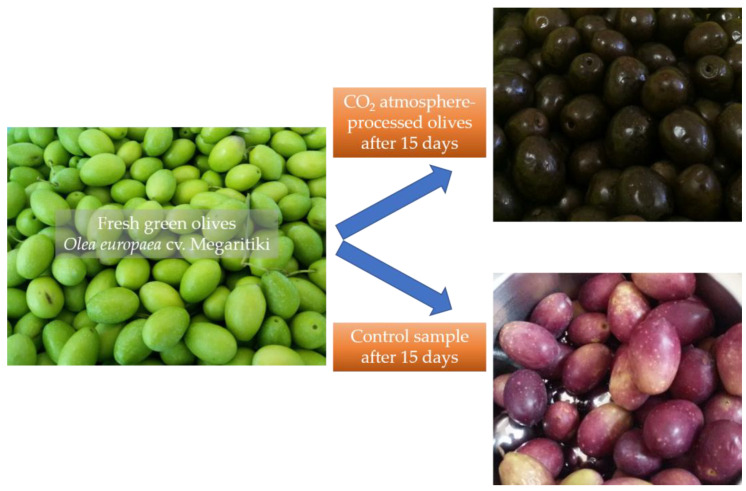
Color changes of olives stored under CO_2_ atmosphere and under regular atmospheric conditions (control), after 15 days.

After 15 days, the CO_2_ debittering procedure was ended and the color of the olives changed from green to brown (Figure 2). The color of the control samples also changed to purple-green. The CO_2_-processed olives presented a 4.11 ± 0.13% loss of moisture, while no significant loss was recorded for the control sample. 

### 3.2. Sensory Evaluation

After 15 days, the samples (both control and CO_2_ atmosphere processed) were subjected to sensory evaluation (Table 1). According to the results, the bitterness of the olives under the CO_2_ atmosphere was significantly decreased (*p* < 0.05). These olives presented the highest acceptability among panelists. Specifically, during the evaluation of the overall preference, 7 out of 12 panelists expressed their preference for the CO_2_ debittered olives, while 5 panelists could not express a clear preference. Dourtoglou et al. [23] proposed that the leafy/neutral aroma of the control olives changed to a distinctive olive flavor with fruity characteristics in the CO_2_-debittered olives. The flavor enhancement could also be attributed to the reduction in bitterness.

### 3.3. Olive Oil Yield

The hardy, dual-purpose Megaritiki olive variety is popular in Greece and produces fruits with medium to high oil yields of high quality [32]. The oil yield from the CO_2_ atmosphere processed olives was 11.14 ± 1.22%, being significantly higher (21%) (*p* < 0.05) than that from the control ones.

### 3.4. Volatile Compounds (VCs)

**Figure 3:**
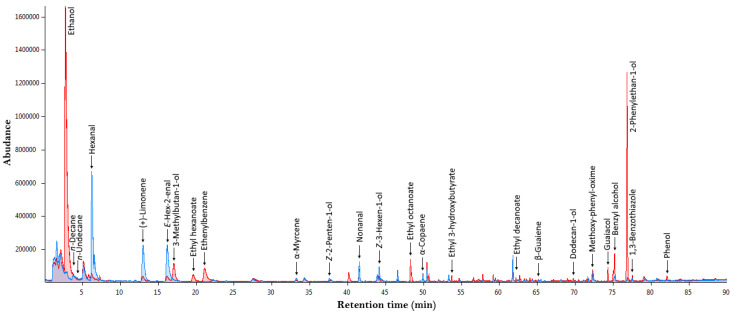
Typical GC-MS chromatogram of the VCs from olive oil storage under CO_2_ atmosphere (red chromatogram) and control sample (blue chromatogram).

The VCs of the oil produced from the control olives and the olives treated with CO_2_ were examined. In Figure 3, typical chromatograms of the VCs are presented, while in Table 2, a comparison of the analyses among the three oils (from CO_2_ treated and control olives, and commercial oil) is presented. 

In total, 30 different VCs, were detected. A total of 18 of them (which account for 83.87 ± 0.60% of the total VCs) were identified in the CO_2_ atmosphere processed sample, followed by 13 in the control (64.83 ± 2.21% of the total VCs) and 11 in the sample of commercial olive oil (56.55 ± 1.76% of the total VCs).

In all cases, aldehydes such as hexanal and *E*-hex-2-enal and alcohols such as *Z*-2-hexen-1-ol and *Z*-3-hexen-1-ol made up the majority of the C_6_ VCs that were found. The primary VCs found were C_6_ compounds, which are produced when linoleic or α-linolenic acids are oxidized under the action of LOX [14]. Variable acyl hydrolase activity and, as a result, good or poor availability of free polyunsaturated fatty acids could be the cause of the different values of C_6_ aldehydes found in samples. 

The oil produced from control olives was characterized by the highest level of hexanal (24.09 ± 1.44%), and *Z*-3-hexen-1-ol (3.22 ± 0.40%). The total C_6_ VCs were 42.93 ± 2.89% (66.22 ± 2.10% of total VCs), and the total C_5_ VCs were 0.69 ± 0.12% (1.06 ± 0.21% of total VCs). Compared to the other samples, *Z*-2-Penten-1-ol was detected only in the control oil (0.69 ± 0.12%). In addition, an aromatic heterocyclic compound, methoxy-phenyl-oxime, was detected only in oil produced from control olives at 1.77 ± 0.08% (its appearance in virgin olive oil was reported earlier [11]). Methoxy-phenyl-oxime is formed during the growth process (fermentation) of *Sorangium cellulosum*, a Gram-negative bacterium of the group myxobacteria [33]. Myxobacteria, which are primarily found in soil, create compounds with known antineoplastic activity [11]. The majority of the hydrocarbons in the waxes that cover olive fruits and leaves are *n*-alkanes. They limit water loss and decrease the cuticle’s ability to become wet [34]. During our study, *n*-alkanes, such as *n*-decane (C_10_) and *n*-undecane (C_11_), were detected only in oil produced from control olives (at 1.75 ± 0.05% and 0.71 ± 0.08%, respectively) because of the slow ripening process. These compounds were detected earlier in virgin olive oils by other authors [35,36,37].

**Figure 4:**
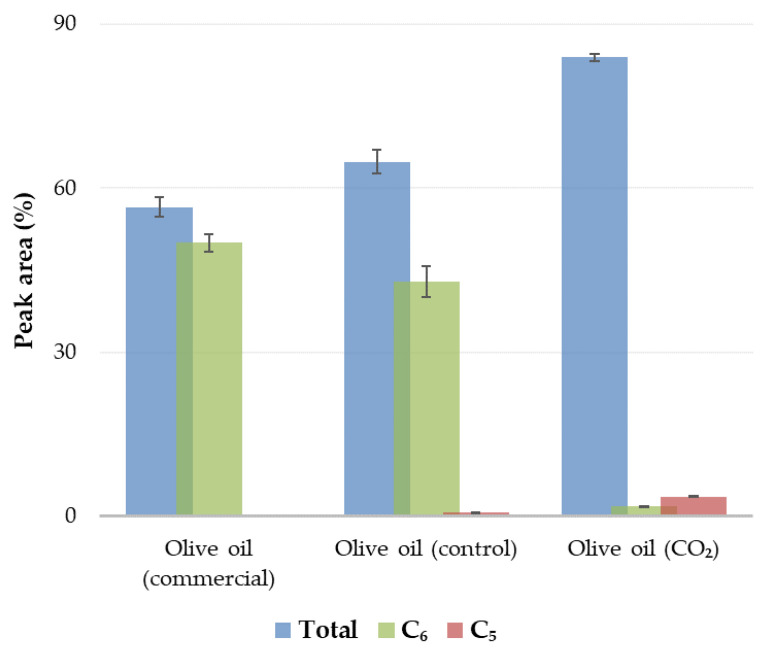
% Peak area of the total, C_6_, and C_5_ VCs in different olive oils.

As regards the commercial oil sample, it was characterized by the highest levels of *E*-hex-2-enal (44.55 ± 1.63%) and *Z*-2-hexen-1-ol (2.76 ± 0.13%). Figure 4 presents, the total C_6_ VCs in the commercial oil which were 50.00 ± 1.62% (88.41 ± 0.12% of total VCs detected). At the same time, no C_5_ VCs were detected in the commercial oil sample. In the commercial oil sample, the green attributes were positively correlated with *Z*-3-hexen-1-ol following the same pattern of the sum C_6_ alcohols and *E,E*-2,4-hexadienal [11]. The *E,E*-2,4-hexadienal has also been detected earlier in olive oil [11,36,38]. *E*-β-Ocimene is also present in virgin olive oil as reported in previous studies [13,35,36,37,39,40] and was detected only in commercial oil sample at a low concentration (0.81 ± 0.16%). α-Copaene is a mono-unsaturated tricyclic sesquiterpene (C_15_). The highest percentage appeared in the commercial oil sample (2.18 ± 0.03%), followed by the oil produced from control olives (0.87 ± 0.09%) and, finally, the oil produced from CO_2_-processed olives (0.55 ± 0.05%). This compound was also reported earlier in virgin olive oil [11,13,35,36,39,40,41]. *E,E*-α-Farnesene a tetra-unsaturated acyclic sesquiterpene (C_15_) was detected only in the commercial oil sample in a low amount (0.91 ± 0.02%). This compound has already been reported to be part of virgin olive oil by other authors [11,21,35,36,39,40,41]. (+)-Cyclosativene, a tetracyclic sesquiterpene (C_15_), was detected during our study only in commercial oil sample (0.25 ± 0.01%). It was also reported to be part of virgin olive oil [11,35,40,41]. Cyclosativene, generated through the sesquiterpene synthase enzyme under the farnesyl pyrophosphate [42], has antioxidant and anticarcinogenic properties [43]. Most terpenes have substantial pharmacological bioactivity due to their anti-inflammatory, anticancer, antidiabetic, antioxidant, or antibacterial activity [44].

In the oil produced from CO_2_ atmosphere-processed olives, only *E*-hex-2-enal (1.05 ± 0.04%) out of the other C_6_ compounds was detected. This happened possibly because *E*-hex-2-enal (the main VC in most European virgin olive oils) decreases (as observed for most of the aldehydes formed from the LOX pathway) with the increase of ripeness [45]. Additionally, ethyl esters such as ethyl 3-hydroxybutyrate (0.28 ± 0.02%) were detected only in oil produced from CO_2_-processed olives. In the biosynthesis of cholesterol, acetoacetate is further reduced to D-3-hydroxybutyrate in the mitochondrial matrix [46]. The fresh green fruity notes are brought on by these C_6_ VCs, which are also present in the aroma of many other vegetable products [15,18]. According to Figure 4, in the oil from CO_2_-processed olives, the total C_6_ VCs were 1.82 ± 0.02% (2.17 ± 0.04% of total VCs). The oil from CO_2_-processed olives greatly differs (*p* < 0.05) from the other oils in the percentage of C_6_ VCs content. The aldehyde and ester levels in the olives, which bestow a pleasant aroma, reduce when olives are stored. VCs that cause bad odors are produced when olives or oil are stored for an extended time [12,20].

C_5_ VCs have a sensory behavior quite similar to that of C_6_ VCs. The level of C_5_ compounds (such as *Z*-2-penten-1-ol) was found to be lower in comparison to the C_6_ compounds. One of the amyl alcohol isomers, 3-methylbutan-1-ol was detected only in the oil produced from CO_2_-processed olives (3.63 ± 0.11%). The *n*-6(S)-hydroperoxylinolenic acid is cleaved anaerobically by the LOX to produce a C_13_-oxoacid (13-oxo-12,9-tridecadienoic acid) and a C_5_ alcohol (*Z*-2-penten-1-ol), according to the study of Salas et al. [18]. An integral component of the olive oil aroma is the C_5_ VCs that bestow fruity and sweet aromas [18]. Hexanal, *E*-hex-2-enal, hexan-1-ol, and 3-methylbutan-1-ol are the main VCs of olive oil that contribute to the positive aroma characteristics (fruity, spicy, and bitter) [14]. In addition, Figure 4 presents the total C_5_ VCs in the oil from CO_2_-processed olives which were 3.63 ± 0.11% (4.33 ± 0.17% of total VCs detected). 

The quality of the oil is also directly correlated with the presence of short-chain alcohols in virgin olive oil. Since small amounts of these alcohols may occur during the ripening of olives, low levels of methanol and ethanol are acceptable. Especially, ethanol has also been detected in low concentrations [12,36,37,47]. On the other hand, significant amounts of ethanol are produced during the fermentation processes, which primarily occur during the storage of olive fruit [48,49]. Additionally, hydroxytyrosol, tyrosol, and ethanol are produced as a result of the hydrolysis of oleuropein during the preservation of olive oil [50]. During our experiments, ethanol was detected only in oil produced from CO_2_-processed olives. It had higher concentrations than reported in previous studies and the highest concentration among the VCs (50.75 ± 2.07%). Substantially, ethanol is a cellular fermentation product from continuous emissions of CO_2_ in olive fruit storage [51].

Aromatic alcohols have been also detected earlier in olive oil [47,52]. Benzyl alcohol and 2-phenylethan-1-ol are constituents of the olive oil volatile fraction [2]. In our case, benzyl alcohol (2.12 ± 0.17%) was detected only in the oil produced from CO_2_-processed olives. 2-Phenylethan-1-ol was detected at a significantly higher level (*p* < 0.05) in oil produced from CO_2_-processed olives (14.69 ± 0.21%) in comparison to the oil produced from control olives (0.54 ± 0.02%). Another odorant, guaiacol (2-methoxyphenol), is already found in green olives but appears in higher concentration as the fruit ripens [52]. Guaiacol and 2-phenylethan-1-ol are the aroma compounds that are important for the fusty flavor [47]. These specific volatile phenols were found in high concentrations in olive oils that had strong fusty, musty, and muddy defects. Their concentration is significantly correlated with the duration of storage and with sensory evaluation, suggesting that they could be used as analytical indices of the oxidation of olive fruits during storage, most likely reflecting the activity of microorganisms [53]. Perhaps this is why it was detected only in oil produced from CO_2_-processed olives but in a very low concentration (0.84 ± 0.13%). Olive-pomace oil contains many more aliphatic alcohols than other olive oils, including dodecan-1-ol [54] and, therefore, it was also detected in oil produced from CO_2_-processed olives in a very low concentration (0.16 ± 0.02%).

Hexanoic acid and ethanol condense to form the ester known as ethanol hexanoate. The formed ethyl hexanoate was detected only in oil produced from CO_2_-processed olives in a low amount (1.47 ± 0.07%). The fatty acid ester ethyl octanoate, sometimes referred to as ethyl caprylate, is created when ethanol reacts with the caprylic acid (a saturated fatty acid labeled 8:0). Ethyl octanoate was detected only in oil produced from CO_2_-processed olives and also in a low percentage (1.46 ± 0.04%). A fatty acid ester called ethyl decanoate, commonly referred as ethyl caprate, is created when ethanol reacts with capric acid (a saturated fatty acid labeled C10:0). Ethyl decanoate was detected only in oil produced from CO_2_-processed olives in a low concentration (0.26 ± 0.07%), as well. These compounds were also detected earlier in virgin olive oil [16,47,52].

Ethenylbenzene, also known as styrene, is a volatile aromatic hydrocarbon that can be produced artificially (industrial paints, adhesives, packages, combustion products) or naturally (plant wax, amino acid fermentation). Ethenylbenzene was detected only in oil produced from CO_2_-processed olives at a low concentration (4.10 ± 0.42%). It has also been reported by other authors as a component of virgin olive oil [38]. Another aromatic heterocyclic compound, 1,3-benzothiazole, was detected in all oils examined in this study at a low percentage (0.42–0.31%). However, no reports on its appearance in olive oils exists in the literature. 1,3-Benzothiazole belongs to benzothiazoles which are a class of high-production volume chemicals with various applications in the industry [55,56]. Benzothiazoles are used in agriculture to prevent and control soil-borne phytopathogenic fungi which affect crops [57].

The measurement of nonanal may be a suitable technique to identify the onset of oxidation. Even if hexanal is present in the original flavor, it was discovered that the hexanal/nonanal ratio is a suitable indicator to identify the start of oxidation and follow its progress [38]. This happened in oil produced from control olives (hexanal/nonanal, 24.09%/1.06%). However, in oil produced from CO_2_-processed olives nonanal was detected at 0.58 ± 0.10%, while hexanal was not detected. The major compounds formed in oxidized olive oil are saturated carbonyl compounds, including pentanal, hexanal, octanal, and nonanal [12], and were not detected in commercial oil. The most frequently appearing volatile markers of oxidation of virgin olive oil during storage are nonanal and *E*-2-decenal [49]. Τhe autoxidation of oleic acid can be exclusively responsible for the appearance of nonanal [58].

Vegetable oil hydrocarbon fraction composition exhibits significant variations that may be used to characterize the oil. Despite their low concentration in olive oil, terpenic hydrocarbons (mono- and sesquiterpenes) exhibit significant variation depending on the variety and the region of production [40]. Limonene is a hydrocarbon classified as a monocyclic monoterpene (C_10_) and generated by alcohol α-terpineol with a loss of a proton. (+)-Limonene smells of oranges, whilst (−)-limonene resembles the smell of lemons [59]. (+)-Limonene was detected in all samples. The highest quantity appeared in oil produced from control olives (13.11 ± 0.70%), followed by commercial oil (1.60 ± 0.06%), while the lowest concentration was detected in the oil produced from CO_2_-processed olives (0.90 ± 0.04%). The presence of (+)-limonene was previously reported in virgin olive oils [21,35,36,37,39,41,52]. α-Myrcene is a structural isomer of β-myrcene and during our study was detected both in oil produced from control olives and commercial oil at the concentration of 0.98 ± 0.12% and 0.50 ± 0.11%, respectively. Virgin olive oil has previously been reported to contain this compound [39,40]. The acyclic monoterpenes β-myrcene (C_10_) and *E*-β-ocimene (C_10_) are generated through the monoterpene synthase enzyme under the geranyl pyrophosphate [59]. β-Guaiene is a bicyclic sesquiterpene (C_15_)—a compound from the azulene group. It was detected only in oil produced from CO_2_-processed olives, in low concentrations (0.12 ± 0.01%). Vichi et al. [40] have identified a different natural chemical compound, δ-guaiene. 

According to the above results, it appears that the oil produced from CO_2_-processed olives presents a much different volatile profile which is attributed to the different procedure of debittering olives. These VCs resulted in the development of a much different flavor, which was highly valued during sensory evaluation by many panelists. Therefore, the storage of olives under the CO_2_ atmosphere can be used not only for their debittering but also for the production of a series of new products with highly valued sensory attributes. It is worth noting that β-guaiene was first detected in an extra virgin olive oil. It is usually found as a component of many plants and mushrooms [44]. Azulene derivatives have been known for centuries for their biological activities and are widely applied in medicine and pharmacy [60]. Guaiene has a delicate, woody flavor and an earthy, spicy aroma [61].

### 3.5. Physicochemical Characteristics

One of the main criteria used for the differentiation among the various varieties of olive oil is acidity, which assesses the free fatty acid (FFA) content. Extra virgin olive oil (EVOO), the most expensive grade of olive oil, is required by Commission Implementing Regulation (EU) No 299/2013 [26] and Commission Delegated Regulation (EU) No 2022/2104 [62] to contain no more than 0.8% FFA. However, all the olive oil characteristics laid down by the Regulation for each category must be met. Three requirements must also be met for olive oil to be certified as EVOO, in addition to the FFA content; it must be manufactured using mechanical extraction techniques without the use of chemicals or hot water, come from first-cold pressing, and have the best flavor possible [63]. In addition, another quality control analysis is the measurement of conjugated dienes and trienes. According to Regulations (EU) No 299/2013 [26] and No 2022/2104 [62], EVOO must not contain more than 2.50 conjugated dienes (K_232_), no more than 0.22 conjugated trienes (K_270_) and the variation of the specific extinction (ΔΚ) must not be more than 0.01. Taking into account the regulations, it was observed (Table 3) that the oil produced from the control sample is out of specification, shows advanced oxidation and is, therefore, of poor quality. The oil produced from CO_2_-processed olives and the commercial oil sample appears to be of good quality and within the legislation requirements for the EVOO category.

Regarding the color measurement, the lightness (L*) of the oil produced from the CO_2_-processed olives was darker than the other samples. Moreover, the oil produced from the control sample had a very light color. In addition, determining the Chroma $\left(C_{ab}^{*}\right)$, i.e., color density, showed that the oil produced from CO_2_-processed olives was less colorful than the other samples. Determination of hue angle ($h_{ab}^{o}$) showed that all samples had a hue of yellow color. In conclusion, the oil produced from CO_2_-processed olives and commercial oil samples appeared to be slightly reddish.

**Figure 5:**
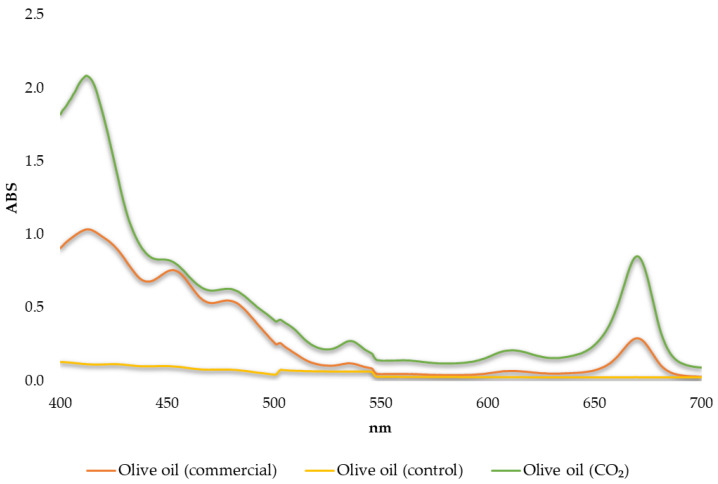
Absorption spectra obtained for chlorophylls (670 nm) and carotenoids (470 nm) in different olive oils.

Moreover, the concentration of total carotenoids and total chlorophylls was evaluated for the determination of pigments. In olive oils, these bioactive substances are responsible for their color [27,64,65,66]. According to the results (Table 3 and Figure 5), it is observed that the oil produced from CO_2_-processed olives had the highest concentration in both these bioactive substances. In Figure 5, the absorption spectra obtained for chlorophylls and carotenoids in different olive oils are presented. The oil produced from control olives appeared to be very poor in these compounds. These results were also verified by the colorimetry technique. Total pigments were measured for the oil produced from the CO_2_-processed sample and were found to be 6.021 ± 0.056 mg/Kg as opposed to the commercial oil sample containing 3.493 ± 0.097 mg/Kg and the oil produced from the control sample (0.782 ± 0.015 mg/Kg).

Subsequently, the oils’ total polyphenol content was determined. The total polyphenols for the oil produced from the CO_2_-processed olives sample were 209.09 ± 18.86 mg of gallic acid equivalents (GAE)/Kg, while for the commercial oil sample was 132.20 ± 8.68 mg GAE/Kg. The oil produced from the control sample was found to have a low content in total polyphenols and specifically 44.52 ± 18.36 mg GAE/Kg. In addition, the determination of α-tocopherol content of the samples was carried out. The concentration of α-tocopherol for the oil produced from the CO_2_-processed sample was 180.89 ± 22.91 mg/Kg, while for the commercial oil sample was 102.04 ± 12.26 mg/Kg. The oil produced from the control sample was found to have a low α-tocopherol concentration of 32.12 ± 5.17 mg/Kg. The Rancimat method was performed to evaluate the oils’ susceptibility to oxidation [67,68]. The oil produced from the CO_2_-processed sample showed an induction period of 13.70 ± 0.14 h, while the commercial oil and the oil produced from control olives samples were 6.35 ± 0.21 and 3.50 ± 0.14 h, respectively. The oxidative stability of the oil deriving from the olives stored under the CO_2_ atmosphere was significantly higher (*p* < 0.05) compared to the two other samples of olive oils.

The fatty acid (FA) profile is shown in Table 4. It is observed that the main FA in all samples was oleic acid (C18:1ω-9c). There were statistically significant differences (*p* < 0.05) in the FA profiles between the different samples. More specifically, monounsaturated fatty acids (MUFAs) were found to be 75.54 ± 0.42, 73.10 ± 0.75, 69.76 ± 0.64%, saturated fatty acids (SFAs) found 15.18 ± 0.18, 13.10 ± 0.09, 17.27 ± 0.40% and polyunsaturated fatty acids (PUFAs) found 9.18 ± 0.13, 6.02 ± 0.09, 12.80 ± 0.21% for the commercial oil, oil produced from control olives and oil produced from CO_2_-processed olives, respectively. Based on the results, regarding olive oils, the overall percentage of FAs and their profile is in accordance with the literature [36,69]. The FAs content of the oils is the primary factor used to establish their quality indices during production, storage, and trading [70]. As a result, the quantity of FAs in oil closely corresponded to the oil’s authenticity and quality.

All the above results indicate that the oil produced from the CO_2_-processed olive sample had all characteristics rendering it suitable to be labeled as EVOO. In addition, the unique organoleptic characteristics such as the aroma, flavor, and color of the product, the high antioxidant activity, and the high content of bioactive compounds (polyphenols, α-tocopherol, carotenoids, and chlorophylls) resulted in an innovative olive oil with excellent chemical characteristics and probable beneficial effects on health [14,65].

## 4. Conclusions

In this study, an alternative procedure for debittering olives was adopted and the derived oil was examined. According to the results, the oil produced from CO_2_-treated olives contains different volatile components, which bestow a unique flavor and aroma to the oil, compared to the control one. Moreover, it was found that the oil deriving from olives stored under CO_2_ exhibits many favorable characteristics. Of utmost importance is its content in antioxidant compounds (i.e., polyphenols), which not only renders the olive oil more stable against oxidation but also grants health benefits upon consumption. This was further evidenced by the oxidative stability test, which validated that the oil derived from olives stored under CO_2_, is two times more stable, compared to the control oil. The proposed procedure enhanced the characteristics of the produced oil, making it comparable to EVOOs. Taking under consideration that the proposed method resulted in the production of olive oil of high quality and of distinct flavor and aroma, it is expected that the results of this study will pave the way for future studies that can revolutionize the olive oil production.

## Figures and Tables

**Table 1:** Sensory assessment of olives stored under a CO_2_ atmosphere and regular atmospheric conditions (control).

Sensory Attributes	Olives (Control)	Olives (CO_2_)
Overall acceptability ^a^	2 (1.0)	8 (0.0)
Bitterness ^b^	9 (0.0)	3 (1.0)
Overall preference ^c^	0	7

^a^ Values represent the scale of overall acceptability (1–9) and the mean and standard deviation (in parentheses) of 12 observations; ^b^ Values represent the scale of bitterness (1–9). Values are the mean and standard deviation (in parentheses) of 12 observations; ^c^ Number of panelists out of 12 who expressed their preference for the particular sample.

**Table 2:** VCs of olive oils.

No.	VCs	CAS Number	RT ^a^	Peak Area (%)		
				Olive Oil (Commercial)	Olive Oil (Control)	Olive Oil (CO_2_)
1.	Ethanol	64-17-5	2.751	ND ^b^	ND	50.75 (2.07) *
2.	*n*-Decane	124-18-5	3.763	ND	1.75 (0.05)	ND
3.	*n*-Undecane	1120-21-4	4.014	ND	0.71 (0.08)	ND
4.	Hexanal ^c^	66-25-1	6.237	ND	24.09 (1.44)	ND
5.	(+)-Limonene	138-86-3	12.944	1.60 (0.06)	13.11 (0.70)	0.90 (0.04)
6.	*E*-Hex-2-enal ^c^	6728-26-3	16.264	44.55 (1.63)	15.62 (1.06)	1.05 (0.04)
7.	3-Methylbutan-1-ol ^d^	123-51-3	17.048	ND	ND	3.63 (0.11)
8.	Ethyl hexanoate	123-66-0	19.617	ND	ND	1.47 (0.07)
9.	Ethenylbenzene	100-42-5	21.112	ND	ND	4.10 (0.42)
10.	*E*-β-Ocimene	3779-61-1	21.570	0.81 (0.16)	ND	ND
11.	α-Myrcene	1686-30-2	33.200	0.50 (0.11)	0.98 (0.12)	ND
12.	*Z*-2-Penten-1-ol ^d^	20273-24-9	37.575	ND	0.69 (0.12)	ND
13.	Nonanal	124-19-6	43.888	ND	1.06 (0.06)	0.58 (0.10)
14.	*Z*-3-Hexen-1-ol ^c^	928-96-1	44.141	1.69 (0.02)	3.22 (0.40)	ND
15.	*E,E*-2,4-Hexadienal ^c^	142-83-6	44.413	1.00 (0.11)	ND	ND
16.	*Z*-2-Hexen-1-ol ^c^	928-94-9	46.586	2.76 (0.13)	ND	ND
17.	(+)-Cyclosativene	22469-52-9	48.232	0.25 (0.01)	ND	ND
18.	Ethyl octanoate	106-32-1	48.282	ND	ND	1.46 (0.04)
19.	α-Copaene	3856-25-5	49.916	2.18 (0.03)	0.87 (0.09)	0.55 (0.05)
20.	Ethyl 3-hydroxybutyrate ^c^	5405-41-4	54.681	ND	ND	0.28 (0.02)
21.	Ethyl decanoate	110-38-3	62.180	ND	ND	0.26 (0.07)
22.	β-Guaiene	88-84-6	65.462	ND	ND	0.12 (0.01)
23.	*E,E*-a-Farnesene	502-61-4	68.114	0.91 (0.02)	ND	ND
24.	Dodecan-1-ol	112-53-8	69.750	ND	ND	0.16 (0.02)
25.	Methoxy-phenyl-oxime	999286-19-2	72.344	ND	1.77 (0.08)	ND
26.	Guaiacol	90-05-1	74.312	ND	ND	0.84 (0.13)
27.	Benzyl alcohol	100-51-6	75.193	ND	ND	2.12 (0.17)
28.	2-Phenylethan-1-ol	60-12-8	76.856	ND	0.54 (0.02)	14.69 (0.21)
29.	1,3-Benzothiazole	95-16-9	77.536	0.31 (0.02)	0.42 (0.02)	0.42 (0.11)
30.	Phenol ^c^	108-95-2	82.123	ND	ND	0.48 (0.04)
Total identified VCs				56.55 (1.76)	64.83 (2.21)	83.87 (0.60)
Total identified C_6_				50.00 (1.62)	42.93 (2.89)	1.82 (0.02)
Total identified C_5_				ND	0.69 (0.12)	3.63 (0.11)

* Values are the mean of triplicate determinations. Standard deviation is given in parentheses; ^a^ Retention time (min); ^b^ Not detected; ^c^ C_6_ VCs; ^d^ C_5_ VCs.

**Table 3:** Physicochemical composition of olive oils.

Physicochemical Parameters	Olive Oil (Commercial)	Olive Oil (Control)	Olive Oil (CO_2_)
*Acidity*			
FFAs (%)	0.68 (0.08) *	0.59 (0.04)	0.51 (0.04)
*Spectrophotometric investigation in the ultraviolet*			
K_232_	2.388 (0.042)	2.870 (0.098)	2.320 (0.038)
K_270_	0.171 (0.008)	1.227 (0.065)	0.156 (0.012)
ΔΚ	0.004 (0.001)	0.133 (0.028)	0.000 (0.000)
*Colorimetry*			
L*	56.9 (0.1)	75.7 (0.6)	37.5 (0.8)
$C_{ab}^{*}$	47.87 (2.76)	48.49 (2.80)	16.85 (1.06)
$h_{ab}^{o}$	88.56 (0.08)	99.32 (0.05)	88.64 (0.09)
*Pigments*			
*C*_carotenoids_ (mg/Kg of oil)	2.613 (0.074)	0.383 (0.004)	3.125 (0.021)
*C*_chlorophylls_ (mg/Kg of oil)	0.881 (0.023)	0.400 (0.012)	2.896 (0.035)
*R*_chl/car_	0.337 (0.001)	1.045 (0.020)	0.927 (0.005)
Total	3.493 (0.097)	0.782 (0.015)	6.021 (0.056)
*Total polyphenols content*			
TPC (mg GAE/Kg of oil)	132.20 (8.68)	44.52 (18.36)	209.09 (18.86)
*α-Tocopherol content*			
α-TC (mg α-tocopherol/Kg of oil)	102.04 (12.26)	32.12 (5.17)	180.89 (22.91)
*Rancimat method*			
Induction time (h)	6.35 (0.21)	3.50 (0.14)	13.70 (0.14)

* Values are the mean of triplicate determinations. The standard deviation is given in parentheses.

**Table 4:** Fatty acid composition of olive oils.

FAMEs		RT ^a^	Peak Area (%)		
			Olive Oil (Commercial)	Olive Oil (Control)	Olive Oil (CO_2_)
C*16:0*	Palmitic	22.018	14.35 (0.05) *	12.47 (0.05)	16.75 (0.25)
C*16:1*	Palmitoleic	22.188	1.39 (0.04)	0.74 (0.04)	2.07 (0.03)
C*18:0*	Stearic	24.368	0.11 (0.03)	0.09 (0.01)	ND ^b^
C*18:1ω-9c*	Oleic	29.027	73.85 (0.35)	72.10 (0.70)	67.45 (0.55)
C*18:2ω-6c*	Linoleic	30.327	8.38 (0.08)	5.45 (0.05)	11.60 (0.30)
C*18:3ω-6*	γ-Linolenic	30.446	ND	ND	0.05 (0.01)
C*18:3ω-3*	α-Linolenic	31.840	0.80 (0.06)	0.55 (0.04)	0.75 (0.07)
C*20:0*	Arachidic	34.115	0.41 (0.05)	0.40 (0.02)	0.33 (0.06)
C*20:1ω-9*	*cis*-11-Eicosenoic	34.893	0.30 (0.03)	0.27 (0.02)	0.24 (0.07)
C*20:2*	*cis*-11,14-Eicosadienoic	35.900	ND	ND	0.08 (0.04)
C*20:3ω-6*	*cis*-8,11,14-Eicosatrienoic	36.945	ND	ND	0.19 (0.06)
C*20:4ω-6*	Arachidonic	37.293	ND	0.03 (0.01)	0.13 (0.02)
C*22:0*	Behenic	38.334	0.20 (0.02)	ND	0.12 (0.06)
C*24:0*	Lignoceric	40.223	0.12 (0.04)	0.15 (0.02)	0.07 (0.04)
	Total identified SFAs		15.18 (0.18)	13.10 (0.09)	17.27 (0.40)
	Total identified MUFAs		75.54 (0.42)	73.10 (0.75)	69.76 (0.64)
	Total identified PUFAs		9.18 (0.13)	6.02 (0.09)	12.80 (0.21)
	Total identified ω-3 FAs		0.80 (0.06)	0.55 (0.04)	0.75 (0.07)
	Total identified ω-6 FAs		8.38 (0.08)	5.47 (0.05)	11.97 (0.24)

* Values are the mean of triplicate determinations. Standard deviation is given in parentheses; ^a^ Retention time (min); ^b^ Not detected.

## Data Availability

All the data is contained within the article.
